# Old Surgical Scar at the Ankle Causing Referred Sciatica-Like Pain Treated With a Scar-Fascial Release Technique

**DOI:** 10.7759/cureus.35090

**Published:** 2023-02-17

**Authors:** Maremi Mizuno, Akio Sakamoto

**Affiliations:** 1 Rehabilitation Unit, Karasuma Hospital, Kyoto, JPN; 2 Department of Orthopaedic Surgery, Kyoto University Hospital, Kyoto, JPN

**Keywords:** osteoarthritis, ankle, active scar, adhesive scar, gait

## Abstract

A surgical scar with adhesions to the underlying fascia and periosteum caused radiating pain to different parts of the scar. Abdominal pain is a common complication of surgical scars, but surgical scar associated with extremity pain is rare. A 75-year-old man had a gait disturbance due to right sciatica-like pain from the thigh to the lower leg for >10 years. He also had mild ankle pain due to osteoarthrosis. The medical history was significant for an ankle injury diagnosed as a sprain and intra-articular small fracture, for which he underwent resection of the bone fragment from the anterior aspect 14 years ago. The surgical scar was adherent to the underlying fascia. The surgical scar was shown to be involved in sciatica-like pain. For the adhesive scar, the scar-fascial release technique was performed by stretching in the direction of the palpated restriction. The sciatica-like pain and gait disturbance resolved one month after the procedure, while the ankle pain remained mild. The current case is a rare case of an adhesive ankle scar causing sciatica-like pain and gait disturbance. The sciatica-like pain involving the proximal lower extremity caused by the ankle scar supports the myofascial meridian concept.

## Introduction

Adhesions are defined as the formation of abnormal fibrous tissue between two anatomically distinct structures. An adhesive scar is defined as restricted skin and soft-tissue movement near the scar, with hyperalgesia, resistance to distraction and movement, tenderness on palpation, and resistance in deeper layers [1,2]. The scar-fascial release aims to restore the stretching of the skin near the scar and normalize the mobility of all soft tissues affected by the scar [2].

Surgical scar adhesions cause not only chronic local pain but also radiating pain [3]. The radiating pain is caused by pulling the adherent tissues with changes in posture in the case of abdominal surgical scars [4]. Although the myofascial meridian concept is widely used in exercise therapy and osteopathic medicine, the scientific basis for the proposed association remains controversial [5].

Adhesive scars with radiating pain can occur after abdominal surgery [2], although pain in the ankle area has not been well-described. The patient reported herein had an adhesive scar in the anterior ankle area and sciatica-like radiating pain. To account for the radiating pain, myofascial meridians are discussed.

## Case presentation

A 75-year-old man (height, 187 cm; weight, 92.5 kg) had right lower extremity pain from the posterolateral thigh to the posterior leg for more than 10 years. The pain worsened with continuous walking. Moreover, the degree of pain progressed year after year. He was unable to walk continuously >100 meters at the time of our initial assessment. When the pain was so severe that he could no longer walk, he would rest while standing, after which the symptoms gradually resolved. The symptoms were assumed to represent intermittent claudication, and the cause of the symptoms was likely neurologic or vascular. The lower extremity symptoms were thought to be sciatic nerve-related at the lumbar spine because plain radiographs showed age-related mild degeneration (data not shown). Vascular claudication due to blood flow dysfunction was unlikely because the anterior tibial artery at the dorsal foot was palpable without lateralization.

**Figure 1:**
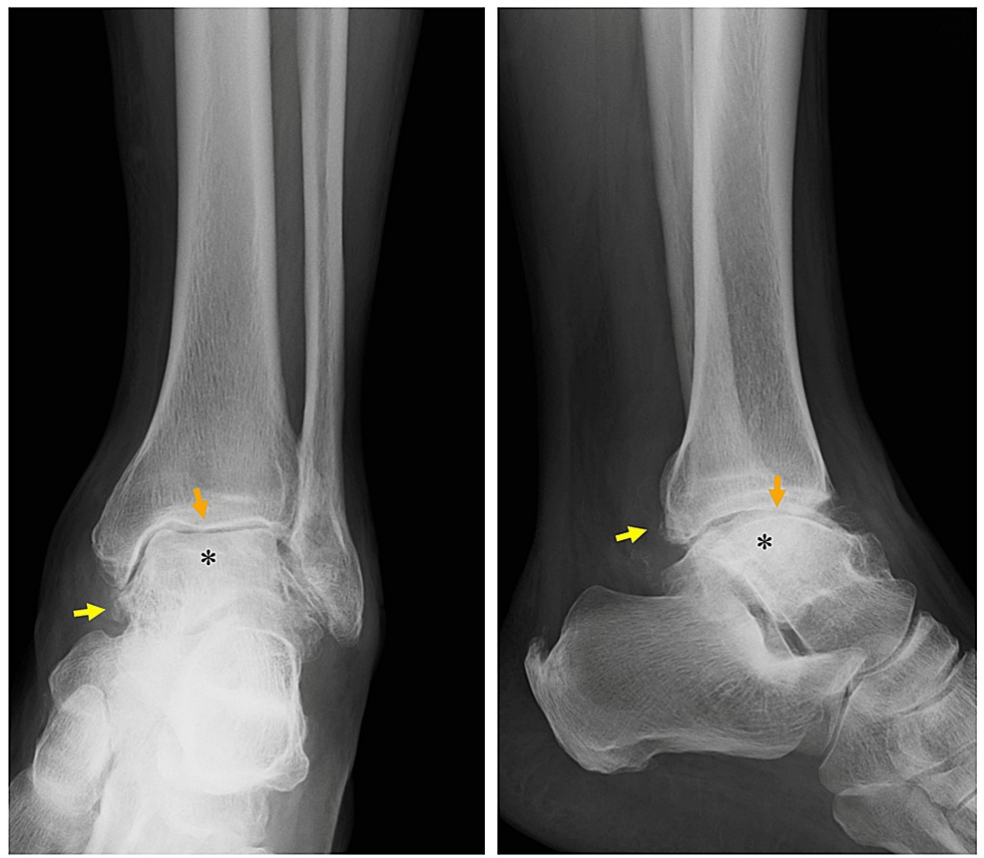
Plain radiograph of the ankle. Osteoarthritis of the ankle is characterized by osteophyte formation (yellow arrows), narrowing of the joint space (orange arrows), and subchondral sclerosis (*) (left: anteroposterior view, right: lateral view).

The patient was prescribed acetaminophen (300 mg four times/day) to manage the pain for more than one year. The physical examination revealed a negative Lasegue test as a sciatic nerve stretch test, although he felt tightness in the right posterolateral thigh. No sensory abnormality was noted based on the pinprick test. Motor function was shown to be normal with manual muscle testing based on a score of 5 in the extremity muscles. Deep tendon reflexes of the patellar and Achilles tendons were normal. He had mild right ankle pain due to osteoarthritis. His ankle injury was diagnosed as a sprain and a small intra-articular fracture. Thus, a bone fragment was removed from the anterior surface 14 years ago at 61 years of age. The medical records for the ankle operation were not available. The range of motion of the right ankle was slightly decreased compared to the left ankle. The plain radiographs of the ankle showed findings of osteoarthritis with narrowing of the joint space and osteophyte formation (Figure 1).

**Figure 2:**
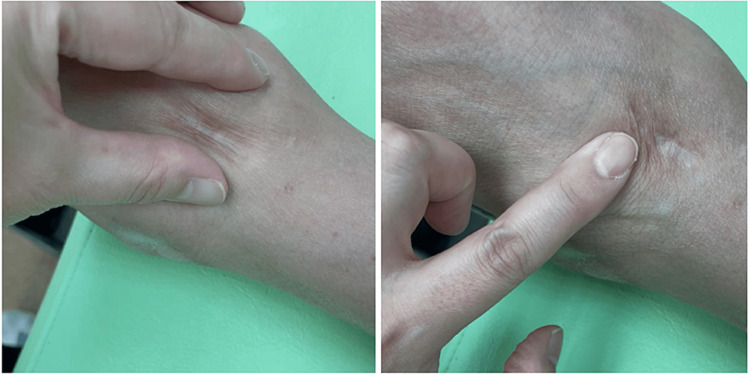
Surgical scar at the anterior ankle (contracture type). Skin extensibility is reduced, in which the skin groove is thickened and tender, when the physical therapist presses medially and laterally (left) and distally (right) around the wound.

A careful physical examination showed that the sciatica-like pain in the extremities originated from the surgical scar involving the anterior ankle, posterior leg, and posterolateral thigh. Therefore, the relationship between the sciatica-like pain in the extremities and the surgical scar was assumed. There was slight tenderness over the scar. The physical examination showed that the surgical scar had no mobility with the tibia, thus the scar was judged to be adherent to the underlying fascia. Skin stretching of the surgical wound was reduced (Figure 2). The sciatica-like pain mentioned by the patient included the surgical scar. The surgical scar was moved according to the movement of the tibialis anterior muscle. Therefore, a relationship between walking, the surgical scar, and sciatica-like pain was assumed. A scar-fascial release technique was initiated by a physical therapist to restore skin extensibility near the scar and normalize all soft-tissue layers affected by the scar. The procedure was applied to deep tissues where the stiffness was felt by palpation. The procedures were continued until release was felt. The treatments occurred once per week in the outpatient unit. Once a day, the patient was instructed to move the scar skin in various directions to the area of restricted skin movement. No apparent improvement in the ankle range of motion was noted. However, one month after treatment, the gait disturbance associated with sciatica-like pain had nearly resolved and he was able to walk >30 minutes without resting, but the mild ankle pain persisted. Aminoacetophenone was no longer needed.

## Discussion

An adhesive scar is described as restricted skin stretching, a thicker and tender skin fold, and resistance to shifting against underlying tissues, including bone [2]. The purpose of soft-tissue scar release techniques is to restore skin stretch close to the scar and make all soft-tissue layers affected by the scar shift normally [2]. In the current case with myofascial tension in an adhesive scar, a myofascial release technique was performed, which successfully ameliorated the symptoms. The scar-fascial release technique appears to be more beneficial for surgical scars than scars after trauma or burns [6]. Although the scar release technique is effective anecdotally, there is limited scientific evidence to support the efficacy [6]; however, surface electromyography of the abdominal and back muscles of a patient with adhesive abdominal surgical scars has been reported [7]. The myofascial release technique at a frequency of once per week for one month was effective in our patient. In previous reports, the frequency of the procedure has been reported as four biweekly sessions for two weeks [4] and 12 sessions two or three times per week for four to eight weeks [2].

Referred pain in patients with abdominal surgical scars occurs with changes in body position that pull on the adhered tissue and cause pain, leading to compensatory protective movement patterns and abnormal posture [4]. The adhesive scar at the ankle in our patient was associated with radiating sciatica-like pain. One explanation for the pain was the neural connection between the innervation of the leg muscle and the sciatic nerve. Another explanation in which the concept of myofascial meridians was applied is widely used in exercise therapy and osteopathic medicine, but the scientific basis for the proposed connection remains controversial [5]. One of the myofascial meridians, a spiral line including the tensor fasciae latae muscle, iliotibial tract, biceps femoris muscle, peroneus longus muscle, and tibialis anterior, has been proposed [5]. In addition, it has been reported that ankle motion leads to fascial force transmission to the posterior aspect of the thigh [8]. These findings may explain the current sciatica-like pain caused by a scar at the ankle.

## Conclusions

The patient reported herein had an adhesive ankle scar causing sciatica-like pain and a gait disturbance. An ankle adhesive scar is a rare anatomic site. The scar-fascial release technique was performed to stretch in the direction of the palpated restriction for the adhesive scar. The myofascial release technique successfully decreased the symptoms. The course of treatment supports the concept of myofascial meridians.
